# Inflammatory and Adipokine Status from Early to Midpregnancy in Arab Women and Its Associations with Gestational Diabetes Mellitus

**DOI:** 10.1155/2021/8862494

**Published:** 2021-01-21

**Authors:** Sara Al-Musharaf, Shaun Sabico, Syed Danish Hussain, Fatima Al-Tawashi, Haifa Bandar AlWaily, Nasser M. Al-Daghri, Philip McTernan

**Affiliations:** ^1^Department of Community Health, College of Applied Medical Science, King Saud University, Riyadh 11451, Saudi Arabia; ^2^Department of Biochemistry, College of Science, King Saud University, Riyadh 11451, Saudi Arabia; ^3^College of Medicine, King Saud University, Riyadh 11451, Saudi Arabia; ^4^School of Science and Technology, Nottingham Trent University, Nottingham NG1 8NS, UK

## Abstract

**Objective:**

To examine differences in maternal serum levels of adipokines (adiponectin, leptin, and resistin) and inflammatory markers (tumor necrosis factor-alpha (TNF-*α*) and interlukin-6 (IL-6)) from early to midpregnancy among Arab women with or without gestational diabetes mellitus (GDM), along with their links to GDM risk.

**Methods:**

This is a multicenter prospective study involving 232 Saudi women attending obstetric care. Both circulating adipokine and markers of inflammation were observed at the first (eight to 12 weeks) and second trimesters (24 to 28 weeks). GDM was screened at 24 to 28 weeks using the International Association of the Diabetes and Pregnancy Study Groups (IADPSG) criteria.

**Results:**

Age and body mass index- (BMI-) matched circulating TNF-*α* was significantly higher in women with GDM in comparison to non-GDM women (*p* = 0.01). Adiponectin and resistin significantly decreased from the first to second trimester in women without GDM (*p* = 0.002 and 0.026, respectively). Leptin presented a significant rise from the first to second trimester in both groups, with a higher increase in women with GDM (*p* = 0.013). Multivariate logistic regression analysis revealed that TNF-*α* was significantly correlated with GDM (*p* = 0.03). However, significance was lost after adjustments for maternal and lifestyle risk factors (OR 23.58 (0.50 to 1119.98), *p* = 0.11).

**Conclusion:**

Inflammatory and adipocytokine profiles are altered in Arab women with GDM, TNF-*α* in particular. Further studies are needed to establish whether maternal inflammatory and adipocytokine profile influence fetal levels in the same manner.

## 1. Introduction

Human pregnancy is linked with physiological states of insulin resistance in different stages of gestation, along with alterations in maternal fat stores [1]. Insulin resistance at the beginning of gestation results from changes in maternal hormones; secretion of placental proinflammatory markers including leptin, resistin, and tumor necrosis factor-alpha (TNF-*α*) [2]; and reduction of anti-inflammatory factors such as adiponectin [3, 4]. Furthermore, systemic inflammation during pregnancy, defined as elevated serum levels of C-reactive protein (CRP) and/or interlukin-6 (IL-6), may serve as a precursor to gestational diabetes mellitus (GDM) [5, 6]. It is suggested that obesity also plays a role in the dysregulation pattern of adipokine production, contributing to a low-grade inflammation status, which enhances insulin resistance and atherogenesis [7]. These factors, along with others, may further enhance pregnancy complications, including GDM [8]. In this context, several meta-analyses have addressed concentrations of adipokines and inflammatory markers in GDM women, proposing that GDM is linked with decreased levels of adiponectin and increased levels of leptin and TNF-*α* [9].

GDM is rising globally [10], with a prevalence of 24.2% in Saudi Arabia alone [11]. Current universal screening for GDM is conducted during the latter half of gestation, which limits time for intervention. Thus, the need for early, simple, and nonfasting screening for women at high risk of GDM is crucial. Detection at early stages will identify women at risk, enabling the opportunity for preventing GDM, and improving maternal and fetal outcomes [12]. The use of clinical risk factors as a screening method has acceptable predictive ability, but these can be enriched by adding markers of inflammation that arise prior to hyperglycemia [13].

Currently, a number of researchers focus their studies on unique biomarkers and their united prediction models [14], since stratification of the risk factors is crucial to point out high-risk women. These individuals will profit most from the application of preventive strategies, such as lifestyle modifications [15], probiotics use [16], and oral hypoglycemic medications [17]. A large number of early pregnancy serum level predictors for GDM have been described in several studies, primarily in small case-control studies. These include glycemic indicators (fasting glucose, postload glucose, and glycated hemoglobin); markers of insulin insensitivity (fasting insulin and sex hormone-binding globulin) [18, 19]; inflammation markers such as CRP [20], TNF-*α* [21], and IL-6 [22]; and adipocyte-derived markers (adiponectin, leptin, and resistin) [23, 24]. However, it is still premature to conclude that these markers can be routinely applied for GDM identification at early stages.

Recent studies have been limited in terms of sample size, confounding risk factors (including race, adiposity, and weight gain), variations in screening, criteria for GDM diagnosis, assay methods used, and GDM reporting [18]. Furthermore, to the best of our knowledge, existing studies have not adjusted for lifestyle factors such as diet, physical activity, and sociodemographics, particularly among the Arabian ethnic group, where the prevalence of GDM is high [11]. Thus, the current study was aimed at investigating differences in adipocytokines and other inflammatory biomarkers among Arab women who either had or did not have GDM during the first and second pregnancy trimesters, and at identifying associations with GDM after controlling for relevant factors.

## 2. Methods

### 2.1. Population and Study Design

The present cohort study involved 232 apparently healthy adult Saudi women during pregnancy (mean age 28.9 ± 5.3 years), who were followed from early (eight to 12 weeks) to midgestation (24 to 28 weeks). Data were collected from the obstetrics clinics where these women received maternal care, including King Fahd Medical City (KFMC), King Khalid University Hospital (KKUH), and Prince Salman Hospital, in Riyadh city. The study was approved by the institutional review boards of KFMC and KKUH. All women signed consent forms prior to inclusion in the study. Inclusion criteria comprised singleton pregnancy with no previous history of diabetes mellitus (type 1 or 2). Exclusion criteria were non-Saudi pregnant women at gestation > 16 weeks, the presence of thyroid or parathyroid disorders, hypertension, malabsorption syndrome, epilepsy, or malignancy. Those receiving long-term medication for any chronic disorder were also excluded from the study.

### 2.2. Data Collection

Anthropometric data included self-reported prepregnancy weight (kg), actual weight during visits, body mass index (BMI, kg/m^2^), midarm circumference (MAC, cm), and waist (cm) to hip (cm) ratio (WHR). Relative body fat percentage (%) was measured from subcutaneous skinfold thickness using a Harpenden caliper (British Indicators, Sussex, England) [25]. Gestational weight gain (GWG) was calculated according to the weight variation between two visits. GWG was presented quantitatively, as the total weight gain from the first to second trimesters, similar to the Institute of Medicine (IOM) GWG guidelines [26].

The questionnaire used in this study was sourced from an epidemiological survey previously published in Saudi Arabia [27]. Socioeconomic data were collected at baseline and at first visit. Participants provided demographic information and clinical history, including the presence of chronic diseases, regularity of menstrual cycle, parity, and previous miscarriages. Lifestyle factors such as dietary intake were measured from a previously used food frequency questionnaire [27], along with multivitamin supplementation questions. Physical activity was assessed using an Arabic version of the International Physical Activity Questionnaire (IPAQ) [28].

Nonfasting blood samples in early pregnancy and fasting blood samples in midpregnancy were collected to measure blood glucose, insulin, HbA1c, and lipid profile (HDL, total cholesterol, and triglycerides), along with adipokines (leptin, resistin, and adiponectin) and inflammatory cytokines (IL-6 and TNF-*α*). During the midpregnancy visit, GDM screening was performed using a glucose tolerance test. Levels of insulin were also assessed. GDM was screened using the International Association of the Diabetes and Pregnancy Study Groups (IADPSG) guidelines, based on one or more insulin values equal to, or exceeding, the following threshold: fasting ≥ 5.1 mmol/l and/or one‐hour postglucose ≥ 10 mmol/l and/or two‐hour postglucose load ≥ 8.5 mmol/l [29].

Serum adipokines (leptin, resistin, and adiponectin) and inflammatory cytokines (IL-6 and TNF-*α*) were quantified using multiplex assay kits, which utilize fluorescent microbead technology, allowing for simultaneous quantification of several target proteins within a single serum sample of 50 *μ*l to 100 *μ*l. These included premixed and fully customized panels that utilize the Luminex xMAP Technology platform (Luminex Corporation, TX, USA). For parameters measured using the multiplex assay, the intra-assay variation was 1.4% to 7.9%, and interassay variation was <21%. Minimum detectable concentrations (MDC) were as follows: leptin, 85.4 pg/ml; adiponectin, 145.4 pg/ml; resistin, 6.7 pg/ml; TNF-*α*, 0.14 pg/ml; and IL-6 (intra- and interassay CV were 7% and 13%, respectively).

Insulin was measured using electrochemiluminescence (ECL) assays (Cobas e411, Roche Diagnostics GmbH, Mannheim, Germany). Serum lipids and glucose were assessed using a routine laboratory chemical analyzer (Konelab, Finland). HbA1c was measured from whole blood using the DCA Vantage Analyzer (Siemens Healthcare, Germany).

### 2.3. Data Analysis

Data analysis was done using SPSS version 21.0 (SPSS, Chicago, IL, USA). Frequencies (%) were used to present categorical variables, and mean and standard deviation were used to present continuous variables. Nonnormal continuous variables were log-transformed prior to parametric testing. An independent sample *t*-test was used to examine significant differences in means between GDM and non-GDM patients. A paired sample *t*-test was used to obtain *p* values for biomarker changes from the first to second trimesters in both GDM and non-GDM groups. Associations between potential risk factors and developing GDM were evaluated using logistic regression adjusted for age and BMI at visit 1, education, parity, previous history of GDM, employment, family history (DM, obesity, and GDM), diet, physical activity, and GWG. *p* value < 0.05 was considered significant.

## 3. Results

### 3.1. Prevalence of GDM

The prevalence of GDM was 42.7% (99/232), based on IADPSG. According to fasting blood glucose, 28.1% (65/232) of participants were identified as GDM and 35.3% (85/232) were identified as GDM using a one-hour or two-hour glucose tolerance test.

### 3.2. Maternal Characteristics

Maternal characteristics of patients are presented in Table 1. No significant differences were observed in patients' anthropometrics between GDM and non-GDM women; however, women in the non-GDM group had significantly higher gestational weight gain as compared to women in the GDM group (*p* = 0.049). Furthermore, women having a past history of GDM were more likely to develop GDM again (*p* < 0.001). In addition, no significant mean differences were observed in the lipid profile of GDM and non-GDM patients. However, patients who developed GDM had significantly higher HbA1c in early pregnancy as compared to women with no GDM (*p* < 0.001).

### 3.3. Adipocytokines, Inflammatory Biomarkers, and Associations

Patients who developed GDM had higher TNF-*α* in midpregnancy (*p* = 0.01) as compared to patients with no GDM. No significant differences were observed in other biomarkers including adiponectin, resistin, IL-6, and leptin in both early and midpregnancies (Table 2). Adiponectin showed an inverse correlation with TG (*r* = −0.23, *p* < 0.01), total cholesterol (*r* = −0.17, *p* < 0.05), and HDL-cholesterol (*r* = −0.14, *p* < 0.05) in early pregnancy. It also showed an inverse correlation with systolic BP (*r* = −0.13, *p* < 0.05) and MAC (*r* = −0.14, *p* < 0.05). In midpregnancy, adiponectin showed a positive association with HOMA-*β* (*r* = 0.25, *p* < 0.05). Resistin in early pregnancy showed a positive correlation with insulin levels in early pregnancy (*r* = 0.17, *p* < 0.05). Leptin in early pregnancy correlated positively with MAC (*r* = 0.16, *p* < 0.05), systolic BP (*r* = 0.15, *p* < 0.05), and insulin (*r* = 0.16, *p* < 0.05). In midpregnancy, leptin correlated positively with fat (*r* = 0.21, *p* < 0.01) and inversely with cholesterol HDL ratio (*r* = −0.16, *p* < 0.05), total cholesterol (*r* = −0.21, *p* < 0.01), and LDL-cholesterol (*r* = −0.22, *p* < 0.01). TNF-*α* and IL-6 were inversely correlated with HDL-cholesterol. Furthermore, TNF-*α* was inversely associated with HOMA-*β* and positively with fasting glucose (Table 3). In multivariate logistic regression analysis, TNF-*α* in midpregnancy appeared to significantly raise the risk of developing GDM in women by 27.81 times (95% CI 1.36-570.22; *p* = 0.031) after controlling for age and BMI (Table 4). However, this significance was lost when controlled for other lifestyle factors.

### 3.4. Inflammatory Markers across Two Visits according to GDM Status

**Figure 1:**
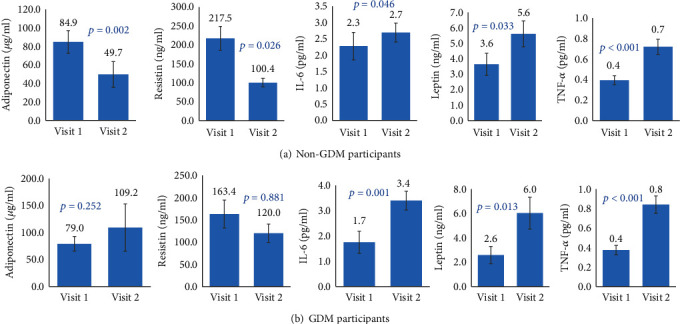
Inflammatory and adipokine markers in (a) non-GDM and (b) GDM patients according to visits.

Inflammatory markers across two visits according to GDM status are presented in Figure 1. When pregnant women were divided into two groups (GDM vs. non-GDM), adiponectin and resistin decreased dramatically from the first to second trimesters in non-GDM women, while in GDM women, both adiponectin and resistin showed a nonsignificant increase. Leptin presented a significant increase from the first to second trimesters in both groups, with a higher increase in the GDM group whilst TNF-*α* and IL-6 showed a significant increase from the first to second trimesters in both groups.

## 4. Discussion

The current study is the first to demonstrate differences and associations of serum adipocytokines and inflammatory biomarkers at early to midterm pregnancy in Arab women with and without GDM. The present study did not reveal any associations between inflammatory and adipocytokine markers from early gestation in the development of diabetes in midgestation. However, TNF-*α* level in midpregnancy was linked to GDM risk, and the significance persisted after adjusting for age and BMI. Hence, the role of TNF-*α* appeared more relevant during midpregnancy in terms of disturbing metabolic profile. Tools for GDM screening in women at high risk can be enhanced by the inclusion of biomarkers representing first trimester inflammation and insulin insensitivity [30, 31].

Placental TNF-*α* has been proposed as a possible facilitator for insulin insensitivity during gestation [32]. Similar to the findings of this study, Syngelaki et al., examining 200 GDM cases and 800 controls, showed that circulating TNF-*α* measured at 11 to 13 weeks gestation did not improve prediction of GDM over maternal characteristics [33]. Thus, TNF-*α* in early pregnancy (<16 weeks) showed no differences in GDM in the present study or in existing studies [32, 33]. Conversely, TNF-*α* in midpregnancy dramatically increased the risk of GDM, even after age and BMI adjustments. It is worth noting here that in all the existing studies noted herein, OGTT was not conducted for all women, but only for those with abnormal random blood glucose at 24 to 28 weeks of gestation. Similar to our results, a study that addressed the prior variable in midpregnancy showed significance with no adjustments [34]. Furthermore, López-Tinoco et al. revealed that TNF-*α* in midpregnancy increased the risk of GDM, even after adjustments for weight, age, and BMI [21]. It is worth noting that after correcting for all maternal and lifestyle risk factors in our study, significance disappeared. This study, to our knowledge, is the only study to have adjusted for all lifestyle factors including parity, education, past GDM, family history, gestational weight gain, diet, and physical activity. Significance may have disappeared due to the small sample size, once all adjustments were made. Other studies did not find a difference between TNF-*α* in GDM samples, compared to non-GDM samples [35], even after adjusting for age and BMI. However, Guillemette et al. and Kirwan et al. propose an association between TNF-*α* and insulin resistance [32, 35]. Existing studies have also found similar results to the current study, which suggests that TNF-*α* is associated positively with fasting glucose and negatively with HOMA-*β* during midpregnancy. This indicates that TNF-*α* may have a role in disturbing GDM markers.

Similar to alterations in insulin sensitivity, studies indicate that adipokines exhibit altered dynamic patterns during normal gestation [36]. As the pregnancy period advances, there are significant variations in adipokines status during gestation [8, 37]. However, each adipokine follows a specific pattern of change during pregnancy [38]. For example, levels of adiponectin progressively decrease [39], while levels of leptin progressively increase during pregnancy [40].

In this study, maternal TNF-*α* and IL-6 showed a significant increase from the first to second trimesters in both groups (GDM vs. non-GDM). The majority of cross-sectional studies show that TNF-*α* levels were raised after the first trimester [41]. Furthermore, when participants were stratified according to GDM presence, adiponectin decreased dramatically from the first to second trimesters in the non-GDM group. This may be explained by the larger sample size for non-GDM women, compared to GDM women. Another explanation may be the higher weight gain and fat percentage in non-GDM compared to GDM women, as GDM women are more actively protected against gaining weight in this cohort. This is in line with literature indicating that adiponectin decreased significantly in normal pregnancy [39]; however, to the best of our knowledge, no studies have precisely investigated adiponectin changes during gestation in women who developed GDM [18]. Leptin presented a significant increase from the first to second trimesters in both groups, with a higher increase in the GDM group. This direction was expected, as leptin concentrations increased from the start of gestation, indicating that a rise in leptin levels are not solely because of maternal weight gain [36]. The placenta plays a role by producing high amounts of leptin messenger RNA and protein. Additionally, leptin receptors are abundant in the placenta [8]. Furthermore, leptin produced from the placenta may contribute to the regulation of fetal growth, independent of maternal glucose levels [41]. This explains why in the current study, gestational weight gain and fat mass were higher in non-GDM vs. GDM women; however, leptin concentrations were elevated in women with GDM compared to non-GDM women, regardless of fat mass, which indicates the placenta's role in producing leptin during pregnancy. Finally, in this study, resistin decreased dramatically from early to midpregnancy in women without GDM, while women with GDM showed an increase in resistin levels from early to midpregnancy. A study that addressed participants with a history of GDM in a previous gestation showed an increase in the concentration of leptin and resistin and a decrease in adiponectin in subsequent gestations [37]. The prior study did not find variations in resistin status between GDM and non-GDM. The higher levels of resistin in non-GDM pregnancy may likely have been due to weight gain and increased adiposity; furthermore, concentrations are thought to rise as the pregnancy term increases, alongside progressive weight gain [36].

The authors acknowledge some limitations to the present study. The first of these is that, after adjusting for all lifestyle factors, we lost significance in the multivariate regression. Second, we did not measure levels of inflammatory and adipokine markers in the third trimester, which may have been able to provide a better view of the behavior of these markers. Third, it appears crucial to differentiate between types of obesity, based on the distribution of fat. In the current study, fat distribution was difficult to assess, as women were pregnant. Studies taking these parameters into account may find major differences. Nevertheless, this study is the first to shed light on the associations of antenatal inflammation with GDM progression in women of Arabian ethnicity. In a systematic review of inflammatory markers and GDM, the need for further studies on inflammation and GDM that include ethnicities other than European or American has been suggested [42]. In this study, inflammatory biomarkers as early indicators of GDM were investigated. To the best of our knowledge, the existing literature is limited by variable adjustments for confounders, small sample size, variations in assay methodology, and standardization. This study was capable of investigating the involvement of medical risk factors for GDM such as age, BMI, past history of GDM, and lifestyle, including diet and physical activity. Furthermore, the majority of existing studies involved either women with previous GDM [43] or obese women [44]; however, the current study adjusted for BMI and previous GDM history and took an approach that compared GDM to controls.

## 5. Conclusion

In conclusion, this study proposes that levels of TNF-*α* are associated with GDM among Arab pregnant women. The expression of inflammatory (TNF-*α* and IL-6) and adipokine profiles among Arab women with GDM are altered, compared to women without GDM. Further studies are needed to investigate whether maternal inflammatory and adipocytokine profile can influence fetal levels in the same manner.

## Figures and Tables

**Table 1:** Maternal characteristics according to GDM status.

Parameters	Non-GDM	GDM	*p* values
*N*	133	99	
Age (years)	30.2 ± 4.5	29.6 ± 5.4	0.39
Prepregnancy BMI (kg/m^2^)	28.4 ± 5.2	28.3 ± 5.7	0.88
BMI (kg/m^2^) at visit 1	29.5 ± 5.9	29.2 ± 5.5	0.69
BMI (kg/m^2^) at visit 2	31.7 ± 5.9	31.1 ± 5.2	0.40
Gestational weight gain (kg)	8.4 ± 5.9	6.6 ± 6.0	0.049
Waist-hip ratio (WHR) visit 1	0.8 ± 0.1	0.9 ± 0.1	0.13
Systolic BP (mmHg)	115.2 ± 15.5	113.1 ± 12.8	0.26
Diastolic BP (mmHg)	68.5 ± 10.9	66.9 ± 9.5	0.26
Body fat (%) visit 1	35.0 ± 4.4	34.3 ± 4.6	0.23
Obstetric and family history			
Multiparous (yes)	78 (58.6)	62 (62.6)	0.54
Caesarean section (yes)	38 (28.5)	26 (26.2)	0.70
Miscarriage (yes)	33 (24.8)	25 (25.2)	0.94
Past GDM (yes)	4 (0.03)	20 (0.20)	<0.001
Biochemical parameters			
HbA1c (%)	5.0 ± 0.5	5.2 ± 0.5	<0.001
Total cholesterol (mmol/l)	5.3 ± 1.0	5.3 ± 1.0	0.68
HDL-cholesterol (mmol/l)	1.4 ± 0.4	1.3 ± 0.4	0.10
Cholesterol-HDL ratio	4.0 ± 1.0	4.2 ± 1.2	0.16
LDL-cholesterol (mmol/l)	3.2 ± 0.7	3.2 ± 0.8	0.83
Triglycerides (mmol/l)	1.5 ± 0.6	1.6 ± 0.6	0.36

Note: data presented as mean ± SD; independent sample *t*-test was used to obtain *p* values; significant at *p* < 0.05.

**Table 2:** Inflammatory markers according to GDM status.

Inflammatory markers			
	Non-GDM	GDM	*p* values
*At visit one*			
Adiponectin (*μ*g/ml)^#^	84.9 ± 75.4	79.0 ± 74.2	0.53
Resistin (ng/ml)^#^	217.5 ± 193.7	163.4 ± 163.4	0.27
IL-6 (pg/ml)^#^	2.3 ± 2.7	1.7 ± 2.4	0.32
Leptin (ng/ml)^#^	3.6 ± 5.1	2.6 ± 4.3	0.22
TNF-*α* (pg/ml)^#^	0.4 ± 0.3	0.4 ± 0.2	0.23

*At visit two*			
Adiponectin (*μ*g/ml)^#^	49.7 ± 85.4	109.2 ± 240.5	0.37
Resistin (ng/ml)^#^	100.4 ± 70.3	120.0 ± 106.1	0.64
IL-6 (pg/ml)^#^	2.7 ± 1.9	3.4 ± 2.0	0.15
Leptin (ng/ml)^#^	5.6 ± 6.0	6.0 ± 7.9	0.78
TNF-*α* (pg/ml)^#^	0.7 ± 0.5	0.8 ± 0.5	0.26

Note: data presented as mean ± SD; # indicates nonnormal variable; independent sample *t*-test was used to obtain *p* values; nonnormal variables were log-transformed prior to parametric testing; *p* value < 0.05 considered significant.

**Table 3:** Correlation between inflammatory markers and other parameters at early and midpregnancy.

Parameters	Visit 1					Visit 2			
	Adiponectin (*μ*g/ml)	Resistin (ng/ml)	IL-6 (pg/ml)	Leptin (ng/ml)	TNF-*α* (pg/ml)	Adiponectin (*μ*g/ml)	IL-6 (pg/ml)	Leptin (ng/ml)	TNF-*α* (pg/ml)
Age (years)					-0.18^∗^				
Systolic BP at V1	-0.13^∗^			0.15^∗^	
MAC at V1	-0.14^∗^			0.16^∗^	
Fat at V1		-0.16^∗^		-0.15^∗^	
Insulin (*μ*U/ml) at V1		0.17^∗^		0.16^∗^	
Total cholesterol (mmol/l) at V1	-0.17^∗^				
Cholesterol-HDL ratio at V1			-0.17^∗^		
Triglycerides (mmol/l) at V1	-0.23^∗∗^				
HDL-cholesterol (mmol/l) at V1	-0.14^∗^				
Fasting glucose (mmol/l) at V2									0.30^∗∗^
Fat at visit 2		-0.34^∗∗^	0.21^∗∗^	-0.27^∗∗^
HDL-cholesterol (mmol/l) at V2		-0.27^∗∗^		-0.35^∗∗^
Cholesterol-HDL ratio at V2		0.17^∗^	-0.16^∗^	0.21^∗^
Total cholesterol (mmol/l) at V2			-0.21^∗∗^	
LDL-cholesterol (mmol/l) at V2			-0.22^∗∗^	
Homa-B at V2	0.25^∗^			-0.21^∗^

Note: data presented as correlation coefficient; ∗∗ and ∗ indicate significance at 0.01 and 0.05, respectively.

**Table 4:** GDM predictors at midpregnancy.

	Unadjusted	Model 1	Model 2
TNF-*α* (pg/ml)^#^	24.4 (1.3 to 464.1)	27.8 (1.4 to 570.2)	23.6 (0.5 to 1120)
*p* value	0.034	0.031	0.11

Note: data are presented as slope, odds ratio (OR), and 95% confidence interval (CI) for OR, using logistic regression analysis, and taking GDM as a dependent variable against potential risk factors at visit 1. Model 1: age and BMI at visit 1; model 2: model 1+parity, education, employment, past GDM, family history (obesity, DM, and GDM), physical activity, diet, and GWG.

## Data Availability

Data available on request.

## Abbreviations

TNF-*α*: Tumor necrosis factor-alpha
IL-6: Interlukin-6
GDM: Gestational diabetes mellitus
GWG: Gestational weight gain
BMI: Body mass index
IADPSG: International Association of the Diabetes and Pregnancy Study Groups
ECL: Electrochemiluminescence
SPSS: Statistical Package for Social Sciences.

## References

[1] Barbour L. A., McCurdy C. E., Hernandez T. L., Kirwan J. P., Catalano P. M., Friedman J. E. (2007). Cellular mechanisms for insulin resistance in normal pregnancy and gestational diabetes. *Diabetes Care*.

[2] Fowler M. (2007). Diabetes: magnitude and mechanisms. *Clinical Diabetes*.

[3] Radaelli T., Uvena-Celebrezze J., Minium J., Huston-Presley L., Catalano P., Hauguel-de M. S. (2006). Maternal interleukin-6: marker of fetal growth and adiposity. *Journal of the Society for Gynecologic Investigation*.

[4] Metzger B. E., Buchanan T. A., Coustan D. R. (2007). Summary and recommendations of the fifth international workshop-conference on gestational diabetes mellitus. *Diabetes Care*.

[5] D'annna R., Baviera G., De Vivo A., Facciola G., Di Benedetto A., Corrado F. (2006). C-reactive protein as an early predictor of gestational diabetes mellitus. *Journal of Reproductive Medicine*.

[6] Yu F., Xue Y., Li C., Shen J., Gao F., Yu Y. (2007). Association of serum interleukin-6 and high-sensitivity C-reactive protein levels with insulin resistance in gestational diabetes mellitus. *Journal of Southern Medical University*.

[7] Freitas Lima L. C., Braga V. d. A., do Socorro de França Silva M. (2015). Adipokines, diabetes and atherosclerosis: an inflammatory association. *Frontiers in Physiology*.

[8] Briana D. D., Malamitsi-Puchner A. (2009). Reviews: adipocytokines in normal and complicated pregnancies. *Reproductive Sciences*.

[9] Xu J., Zhao Y. H., Chen Y. P. (2014). Maternal circulating concentrations of tumor necrosis factor-alpha, leptin, and adiponectin in gestational diabetes mellitus: a systematic review and meta-analysis. *The Scientific World Journal*.

[10] Ferrara A. (2007). Increasing prevalence of gestational diabetes mellitus: a public health perspective. *Diabetes Care*.

[11] Wahabi H., Fayed A., Esmaeil S., Mamdouh H., Kotb R. (2017). Prevalence and complications of pregestational and gestational diabetes in Saudi women: analysis from Riyadh Mother and Baby cohort study (RAHMA). *BioMed Research international*.

[12] Harrison C., Lombard C., East C., Boyle J., Teede H. (2015). Risk stratification in early pregnancy for women at increased risk of gestational diabetes. *Diabetes Research and Clinical Practice*.

[13] Correa P. J., Vargas J. F., Sen S., Illanes S. E. (2014). Prediction of gestational diabetes early in pregnancy: targeting the long-term complications. *Gynecologic and Obstetric Investigation*.

[14] Poon L. C., McIntyre H. D., Hyett J. A., da Fonseca E. B., Hod M., FIGO Pregnancy and NCD Committee (2018). The first-trimester of pregnancy - A window of opportunity for prediction and prevention of pregnancy complications and future life. *Diabetes Research and Clinical Practice*.

[15] Rönö K., Stach-Lempinen B., Klemetti M. M. (2014). Prevention of gestational diabetes through lifestyle intervention: study design and methods of a Finnish randomized controlled multicenter trial (RADIEL). *BMC Pregnancy and Childbirth*.

[16] Nitert M. D., Barrett H. L., Foxcroft K. (2013). SPRING: an RCT study of probiotics in the prevention of gestational diabetes mellitus in overweight and obese women. *BMC Pregnancy and Childbirth*.

[17] Haw J., Galaviz K. I., Straus A. N. (2017). Long-term sustainability of diabetes prevention approaches: a systematic review and meta-analysis of randomized clinical trials. *JAMA Internal Medicine*.

[18] Abell S. K., De Courten B., Boyle J. A., Teede H. J. (2015). Inflammatory and other biomarkers: role in pathophysiology and prediction of gestational diabetes mellitus. *International Journal of Molecular Sciences*.

[19] Maged A. M., Moety G. A. F., Mostafa W. A., Hamed D. A. (2014). Comparative study between different biomarkers for early prediction of gestational diabetes mellitus. *The Journal of Maternal-Fetal & Neonatal Medicine*.

[20] Qiu C., Sorensen T. K., Luthy D. A., Williams M. A. (2004). A prospective study of maternal serum C-reactive protein (CRP) concentrations and risk of gestational diabetes mellitus. *Paediatric and Perinatal Epidemiology*.

[21] López-Tinoco C., Roca M., Fernández-Deudero A. (2012). Cytokine profile, metabolic syndrome and cardiovascular disease risk in women with late-onset gestational diabetes mellitus. *Cytokine*.

[22] Hassiakos D., Eleftheriades M., Papastefanou I. (2015). Increased maternal serum interleukin-6 concentrations at 11 to 14 weeks of gestation in low risk pregnancies complicated with gestational diabetes mellitus: development of a prediction model. *Hormone and Metabolic Research*.

[23] Lacroix M., Battista M.-C., Doyon M. (2013). Lower adiponectin levels at first trimester of pregnancy are associated with increased insulin resistance and higher risk of developing gestational diabetes mellitus. *Diabetes Care*.

[24] Powe C. E. (2017). Early pregnancy biochemical predictors of gestational diabetes mellitus. *Current Diabetes Reports*.

[25] Durnin J., Womersley J. (1974). Body fat assessed from total body density and its estimation from skinfold thickness: measurements on 481 men and women aged from 16 to 72 years. *The British Journal of Nutrition*.

[26] Rasmussen K. M., Catalano P. M., Yaktine A. L. (2009). New guidelines for weight gain during pregnancy: what obstetrician/gynecologists should know. *Current Opinion in Obstetrics & Gynecology*.

[27] Al-Musharaf S., Al-Othman A., Al-Daghri N. M. (2012). Vitamin D deficiency and calcium intake in reference to increased body mass index in children and adolescents. *European Journal of Pediatrics*.

[28] Mohd M. M. A.-H., Phung H., Sun J., Morisky D. E. (2016). The predictors to medication adherence among adults with diabetes in the United Arab Emirates. *Journal of Diabetes & Metabolic Disorders*.

[29] Panel IADPSG Consensus (2010). International association of diabetes and pregnancy study groups recommendations on the diagnosis and classification of hyperglycemia in pregnancy. *Diabetes Care*.

[30] Georgiou H. M., Lappas M., Georgiou G. M. (2008). Screening for biomarkers predictive of gestational diabetes mellitus. *Acta Diabetologica*.

[31] Smirnakis K. V., Plati A., Wolf M., Thadhani R., Ecker J. L. (2007). Predicting gestational diabetes: choosing the optimal early serum marker. *American Journal of Obstetrics & Gynecology*.

[32] Kirwan J. P., Hauguel-De Mouzon S., Lepercq J. (2002). TNF-*α* is a predictor of insulin resistance in human pregnancy. *Diabetes*.

[33] Syngelaki A., Visser G. H., Krithinakis K., Wright A., Nicolaides K. H. (2016). First trimester screening for gestational diabetes mellitus by maternal factors and markers of inflammation. *Metabolism-Clinical and Experimental*.

[34] Gao X. L., Yang H. X., Zhao Y. (2008). Variations of tumor necrosis factor-*α*, leptin and adiponectin in mid-trimester of gestational diabetes mellitus. *Chinese Medical Journal*.

[35] Guillemette L., Lacroix M., Battista M.-C. (2014). TNF*α* dynamics during the oral glucose tolerance test vary according to the level of insulin resistance in pregnant women. *The Journal of Clinical Endocrinology & Metabolism*.

[36] Miehle K., Stepan H., Fasshauer M. (2012). Leptin, adiponectin and other adipokines in gestational diabetes mellitus and pre-eclampsia. *Clinical Endocrinology*.

[37] Guelfi K. J., Ong M. J., Li S. (2017). Maternal circulating adipokine profile and insulin resistance in women at high risk of developing gestational diabetes mellitus. *Metabolism - Clinical and Experimental*.

[38] Lain K. Y., Daftary A. R., Ness R. B., Roberts J. M. (2008). First trimester adipocytokine concentrations and risk of developing gestational diabetes later in pregnancy. *Clinical Endocrinology*.

[39] Catalano P., Hoegh M., Minium J. (2006). Adiponectin in human pregnancy: implications for regulation of glucose and lipid metabolism. *Diabetologia*.

[40] Schubring C., Englaro P., Siebler T. (1998). Longitudinal analysis of maternal serum leptin levels during pregnancy, at birth and up to six weeks after birth: relation to body mass index, skinfolds, sex steroids and umbilical cord blood leptin levels. *Hormone Research in Paediatrics*.

[41] Fasshauer M., Blüher M., Stumvoll M. (2014). Adipokines in gestational diabetes. *The Lancet Diabetes & Endocrinology*.

[42] Gomes C. P., Torloni M. R., Gueuvoghlanian-Silva B. Y., Alexandre S. M., Mattar R., Daher S. (2013). Cytokine levels in gestational diabetes mellitus: a systematic review of the literature. *American Journal of Reproductive Immunology*.

[43] Bellos I., Fitrou G., Pergialiotis V., Perrea D. N., Daskalakis G. (2019). Serum levels of adipokines in gestational diabetes: a systematic review. *Journal of Endocrinological Investigation*.

[44] Thagaard I. N., Krebs L., Holm J.-C., Lange T., Larsen T., Christiansen M. (2017). Adiponectin and leptin as first trimester markers for gestational diabetes mellitus: a cohort study. *Clinical Chemistry and Laboratory Medicine (CCLM)*.

